# Prospective evaluation of clarithromycin in recurrent chronic rhinosinusitis with nasal polyps

**DOI:** 10.1016/j.bjorl.2019.09.008

**Published:** 2019-11-02

**Authors:** Thiago Freire Pinto Bezerra, Rogério Pezato, Pâmella Marletti de Barros, Larissa Leal Coutinho, Leidianny Firmino Costa, Fabio Pinna, Richard Voegels

**Affiliations:** a Universidade Federal de Pernambuco (UFPE), Hospital das Clínicas, Departamento de Otorrinolaringologia, Recife, PE, Brazil; b Faculdade de Medicina da Universidade de São Paulo (FMUSP), São Paulo, SP, Brazil; c Escola Paulista de Medicina (EPM/Unifesp), São Paulo, SP, Brazil; d Instituição Materno Infantil de Pernambuco (IMIP), Departamento de Otorrinolaringologia, Recife, PE, Brazil

**Keywords:** Sinusitis, Clarithromycin, Nasal polyp, Treatment

## Abstract

**Introduction:**

The antiinflammatory effects of macrolides, especially clarithromycin, have been described in patients with chronic rhinosinusitis without polyps and also other chronic inflammatory airway diseases. There is no consensus in the literature regarding the effectiveness of clarithromycin in patients with chronic rhinosinusitis with sinonasal polyposis and the national literature does not report any prospective studies on the efficacy of clarithromycin in chronic rhinosinusitis in our population.

**Objective:**

To evaluate the effect of clarithromycin in the adjunctive treatment of recurrent chronic rhinosinusitis with sinonasal polyposis refractory to clinical and surgical treatment.

**Methods:**

Open prospective study with 52 patients with chronic rhinosinusitis and recurrent sinonasal polyposis. All subjects received nasal lavage with 20 mL 0.9% SS and fluticasone nasal spray, 200 mcg / day, 12/12 h for 12 weeks; and clarithromycin 250 mg 8/8 h for 2 weeks and, thereafter, 12/12 h for 10 weeks. The patients were assessed by SNOT 20, NOSE and Lund-Kennedy scales before, immediately after treatment and 12 weeks after treatment. The patients were also evaluated before treatment with paranasal cavity computed tomography (Lund-Mackay) and serum IgG, IgM, IgA, IgE and eosinophil levels. The outcomes evaluated were: SNOT-20, NOSE and Lund-Kennedy.

**Results:**

Most patients were women, aged 47 (15) years (median / interquartile range), and 61.5% (32/52) had asthma. All patients completed the follow-up after 12 weeks and 42.3% (22/52) after 24 weeks. Treatment resulted in a quantitative decrease in the SNOT-20 [2.3 (1.6) vs. 1.4 (1.6); Δ = −0.9 (1.1); *p* < 0.01]; NOSE [65 (64) vs. 20 (63); Δ = −28 (38), *p* < 0.01] and Lund-Kennedy [11 (05) vs. 07 (05); Δ = −2 (05); *p* < 0.01] scores. SNOT-20 showed a qualitative improvement (>0.8) in 54% (28/52, *p* < 0.04) of patients, a group that showed lower IgE level [108 (147) vs. 289 (355), *p* < 0.01]. The group of patients who completed follow-up 12 weeks after the end of treatment (n = 22) showed no worsening of outcomes.

**Conclusion:**

Long-term adjuvant use of low-dose clarithromycin for chronic rhinosinusitis patients with recurrent sinonasal polyposis refractory to clinical and surgical treatment has resulted in improved quality of life and nasal endoscopy findings, especially in patients with normal IgE levels. This improvement persisted in the patient group evaluated 12 weeks after the end of the treatment.

## Introduction

Rhinosinusitis (RS) is one of the most common complaints reported at medical appointments in the United States, and one of the main reasons for the prescription of antibiotics and loss of work productivity. Approximately 135 out of every 1000 Americans or a total of 31 million people are affected in the United States each year, at a total cost of US$ 6 billion dollars a year.^1^, ^2^, ^3^ The RS are divided into acute (bacterial), chronic with polyps, chronic without polyps and allergic fungal rhinosinusitis.^4^ Chronic Rhinosinusitis (CRS) with Nasal Polyps (CRSwNP) is defined by clinical and endoscopic criteria. The patient should report two or more of the following symptoms for more than 12 weeks, and one of these should be one of the first two: a) Nasal blockage or congestion; b) Anterior nasal discharge or posterior nasal drip; c) facial pain or pressure; d) Reduced or absent sense of smell.^5^ Nasal endoscopy should show bilateral nasal polyposis.^5^, ^6^ One group of these patients have recurrent CRSwNP, defined by the absence of remission after sinusectomy performed more than 6 months before and continuous postoperative drug treatment with topical corticosteroid nasal spray associated with isotonic nasal saline solution.^5^ There are some treatment alternatives for this group of patients, but none are effective for all cases.

Macrolides (MAC) are an anti-inflammatory option for the treatment of CRS. Although the first publication about the prescription of this class of drugs as an antibiotic agent by Kataura et al. occurred in 1965, the first publication of its use as an anti-inflammatory agent in chronic upper airway diseases occurred in 1993.^7^ No bactericidal effect would be associated with their action in these chronic diseases, as maximum serum levels during therapy would be below the minimum inhibitory concentration of the clinically isolated bacteria,^8^ and treatment is effective in patients infected with strains of MAC-resistant bacteria, such as *Pseudomonas aeruginosa*.^9^ Zeng et al. showed that Clarithromycin (CLA) had a dexamethasone-like anti-inflammatory effect in patients with CRS: increase in anti-inflammatory mediators and decrease in the production of Th1 and Th2 response of the sinonasal mucosa.^10^ Although the use of clarithromycin by patients with CRSwNP is controversial, recent studies have shown a much superior outcome in prolonged use of this drug for patients with CRSsNP. However, recent studies have shown the diversity of endotypes in populations of patients with CRSwNP, and we are not aware of the distribution of these endotypes in Brazil.^11^

Open studies with a control group have recently shown the effect of prolonged clarithromycin use in patients with CRSwNP, aimed at preventing recurrence soon after the sinusectomy.^12^ There are no studies in the literature analyzing the effect of CLA on the quality of life of patients with CRSwNP in Brazil.

## Objective

To evaluate the effect of low-dose clarithromycin for 12 weeks on the quality of life of patients with recurrent chronic rhinosinusitis with sinonasal polyposis refractory to clinical and surgical treatment.

## Method

This was an open, prospective, self-paired study carried out in a tertiary hospital in the city of São Paulo, from 2011 to 2012 after approval by the Research Ethics Committee (0523/08). Patients aged 18 years of age or older, with recurrent CRS, defined by the absence of CRS remission after sinusectomy performed more than six months before and continuous postoperative drug treatment with topical corticosteroid nasal spray associated with isotonic nasal saline solution were included.

The medication used throughout the year after surgery was nasal irrigation with 20 mL of 0.9% saline solution in each nostril twice daily and fluticasone propionate, nasal spray, 100 mcg in each nostril 2 times daily. For 3 months before the beginning, and during the study period, the patients were instructed not to use systemic corticosteroids, local or systemic antibiotics.

The exclusion criteria were: secondary causes of CRS; septal perforation; history of nasal trauma or fractures; primary or secondary immune deficiencies; craniofacial syndrome; pregnant women; nasal granulomatous disease; malignant tumors; post-radiotherapy of the head and neck.

### Study protocol

A total of 52 consecutive patients treated during the study period were included. All added the use of CLA to the therapeutic regimen with the doses described above for nasal irrigation with saline solution and fluticasone nasal spray. CLA dose was 250 mg orally every 8 h for 2 weeks and, thereafter, 250 mg orally every 12 h for 10 weeks. Initial assessments included complete anamnesis, quality of life assessment with SNOT-20 and NOSE questionnaires, nasal endoscopy (classified according to the Lund-Kennedy criteria), computed tomography of the paranasal cavities (classified according to the Lund-Mackay criteria) and serum measurements of IgG, IgM, IgA, IgE and eosinophils. In case of uncertainty when filling out the quality of life questionnaires, such as using the scale or regarding symptom-related terms, an attending physician without knowledge of the previous endoscopic examination and prior to the new endoscopic examination, assisted by helping with the understanding of the forms, without influencing the results. The patients were also investigated regarding the concomitant diagnosis of rhinitis, according to the skin-prick test and specific IgE; asthma, according to American Thorax Society criteria; and AERD, according to clinical history.

All patients completed the evaluation after 3 months of treatment (12 weeks) and 43.4% (22/52) completed the 3-month follow-up after treatment completion (24 weeks). The outcomes evaluated after treatment (12 weeks) and 12 weeks after the end of treatment (24 weeks) comprised the qualitative and quantitative improvement of SNOT-20 score, quantitative improvement of NOSE and Lund-Kennedy score, and magnitude of effect. The qualitative improvement of SNOT-20 was defined by a reduction >0.8 in the total SNOT-20 score.

Patients were also assessed using an exploratory approach for the association between outcome and preoperative assessment. Side effects were explained in the informed consent form and at each assessment during the study, patients were asked about the appearance of side effects.

### Statistical analysis

Wilcoxon's *t* test was used to compare the values. Dichotomous data were analyzed by Chi-square or Fisher's exact test. The binomial test was used to evaluate the qualitative improvement in SNOT-20. A *p* value <0.05 was considered statistically significant. The magnitude of effect at 12 weeks after the beginning of treatment was compared with previously published standardizations: 0.2; 0.5; 0.8 represented, respectively, a slight, a moderate and high improvement.

## Results

The median age was 47 years (interquartile range 15), most were women (27/25) and 61.5% (32/52) had asthma (Table 1). All patients completed the 12-week follow-up and 22 patients completed the 24-week follow-up.

**Table 1:** Data from the study patients.

Variables	n = 52
Age	47 (15)^a^
Gender (M/F)	0.93 (25/27)
Asthma	61.5% (32/52)
AERD	36.5% (20/52)
Rhinitis	30.8% (16/52)

M, Male; F, Female; AERD, Aspirin-exacerbated respiratory disease.

a Median (interquartile interval).

CLA treatment resulted in a quantitative decrease in the SNOT-20 [2.3 (1,6) *vs*. 1.4 (1,6); *p* < 0.01]; NOSE [65 (64) *vs*. 20 (63); *p* < 0.01] and Lund-Kennedy scores [11 (05) *vs*. 07 (05); *p* < 0.01], after 12 weeks of treatment. It was also demonstrated that 53.8% (28/52) of the patients had a qualitative improvement in the SNOT-20 questionnaire (*p* < 0.04).

In the group of patients who completed the 12-week follow-up after the end of the treatment (n = 22), there was no statistically significant alterations in SNOT-20 and NOSE scores after 12 weeks of drug withdrawal, but a decrease in Lund-Kennedy score was observed [7 (5) *vs*. 5 (8); *p* < 0.01]. The magnitude of effect on SNOT-20 was 0.81 and on NOSE was 0.83 (Table 2, Table 3).

**Table 2:** Outcomes after clinical treatment with clarithromycin - median (interquartile range).

	Pre-treatment	Post-treatment	Improvement after treatment
SNOT-20	2.3 (1.6)	1.4 (1.6)	−0.9 (1.1)
Clinically significant improvement in SNOT-20			53.8% (28/52)
NOSE	65 (64)	20 (63)	−28 (38)
Lund-Kennedy	11 (05)	07 (05)	−2 (05)

**Table 3:** Sustained improvement assessment after treatment completion.

Variables	Improvement after treatment	*P*	Improvement 12 weeks after treatment completion	*P*
SNOT-20	−0.9 (1.1)	<0.01^a^	−0.18 (−0.78)	0.81
NOSE	−28 (38)	<0.01^a^	0 (28)	0.23
Lund-Kennedy	−2 (5)	<0.01^a^	−2 (4)	<0.01^a^

a Median (interquartile interval).

When evaluating the pretreatment data most associated with clinical improvement after CLA use, a lower IgE level was observed [108 (147) vs. 289 (355); *p* < 0.01) when compared to patients without CLA response. Higher serum IgG [1279 (294) vs. 1044 (380); *p* < 0.01)] and IgM measurements [146 (77) vs. 99 (81); *p* < 0.01)] were also demonstrated in the patients who showed a clinically significant improvement (Table 4).

**Table 4:** Results of preoperative laboratory tests according to clinically significant improvement in SNOT-20 (score decrease > 0.8).

Variables	Overall	Clinically significant improvement in SNOT-20	Absence of clinically significant improvement in SNOT-20	*p*
Eosinophils	300 (500)	400 (475)	300 (275)	0.138
% eosinophils	5 (4.4)	6.3 (3.9)	4.1 (4.4)	0.08
IgA^a^	280 (110)	267 (106)	313 (119)	0.24
IgE^a^	137 (370)	108 (147)	289 (355)	0.01
IgG	1198 (427)	1279 (294)	1044 (380)	<0.01
IgM^a^	121 (95)	146 (77)	99 (81)	<0.01
VHS	5 (07)	7 (06)	3 (03)	0.21

a Statistically significant.

In the pre-treatment assessment, no difference was observed between the groups regarding the presence of asthma, AERD, rhinitis, SNOT-20, NOSE, Lund-Kennedy, or Lund-Mackay scores (Table 5, Table 6).

**Table 5:** Preoperative variables according to Clinically Significant Improvement (CSI) in SNOT-20 (score decrease >0.8).

Variables	Overall	CSI present	CSI absent	*p*
Age	47 (15)	46 (14)	50.5 (16)	0.6
Gender (M/F)	0.93 (25/27)	0.47 (9/19)	2 (16/08)	0.01
Asthma	61.5%	60.7%	62.5%	0.89
AERD	36.5%	35.5%	37.5%	0.89
Rhinitis	30.8%	35.7%	25%	0.4
SNOT-20	2.3 (1.6)	2.72 (1.6)	2.18 (1.64)	0.20
NOSE	65 (64)	70 (56)	63 (68)	0.31
Lund-Kennedy	11 (05)	11 (05)	10 (07)	0.77
Lund-Mackay	18 (07)	16 (09)	18 (03)	0.302

**Table 6:** Pretreatment assessment according to the clinical response regarding the presence of asthma, AERD, rhinitis, SNOT-20, NOSE, Lund-Kennedy or Lund-Mackay scores.

Variables	General	Response	No response	*p*
Age	47 (15)	46 (14)	50.5 (16)	0.6
Gender (M:F)	0.93 (25/27)	0.47 (9/19)	2 (16/08)	0.01
Asthma	61.5%	60.7%	62.5%	0.89
AERD	36.5%	35.5%	37.5%	0.89
Rhinitis	30.8%	35.7%	25%	0.4
SNOT-20	2.3 (1.6)	2.72 (1.6)	2.18 (1.64)	0.02
NOSE	65 (64)	70 (56)	63 (68)	0.31
Lund-Kennedy	11 (05)	11 (05)	10 (07)	0.77
Lund-Mackay	18 (07)	16 (09)	18 (03)	0.302

## Discussion

Long-term use of low-dose CLA resulted in an improvement in the disease-specific quality of life related to rhinosinusitis (SNOT-20), nasal obstruction (NOSE), and nasal endoscopy (Lund-Kennedy). There was no symptom worsening in the group of patients who completed the 3-month follow-up after the end of the treatment (n = 22).

Topical corticosteroid nasal spray constitutes the first treatment option for patients with CRSwNP, supported by high level of evidence. However, a significant number of patients do not have a satisfactory response to this drug. EPOS defines patients as having difficult-to-control CRSwNP when they have no symptom resolution after surgery followed by corticosteroid nasal spray, requiring at least two short courses of systemic antibiotics or corticosteroids in the previous year.^5^

These patients may need an alternative treatment, preferably non-surgical. Our study shows that in patients with CRScNP and low IgE levels, CLA is an option associated with a good outcome, resulting in a satisfactory response in more than half of patients.

The mechanism of action of prolonged MAC therapy remains an open question. Previous investigations have focused on its several anti-inflammatory properties, such as suppressing neutrophil exudation in tissue^13^ and fibroblast proliferation in nasal polyps;^14^ inhibition of the proliferative response of CD4 + T lymphocytes in a similar manner as that found for prednisolone; and suppression of antigen-specific immune response of dendritic cells.^14^, ^15^ In the cytokines at the inflammation site, it causes a reduction in IL-8,^16^ RANKL, GM-CSF,^17^ AP-1,^18^ IFN-γ, ICAM-1 and IgE.^15^, ^19^, ^20^ It also reduces DNA synthesis in the endotoxin-exposed nasal epithelium,^21^ decreases the secretion of water and mucus through the nasal mucosa,^22^ improves mucociliary clearance in patients with sinus-bronchial syndrome and rheological properties of nasal mucus,^23^ in addition to inhibiting the formation of *Pseudomonas aeruginosa* biofilm and MUC5AC transcription induced by TNF-α.^24^

The effectiveness of one macrolide that has been previously studied for CRS, azithromycin, is controversial. Videler et al. showed in a clinical trial the absence of significant benefit regarding the use of azithromycin for patients with CRS without differentiating for the presence of polyps.^25^ However, another clinical trial showed better results with the use of this medication during the postoperative period.^26^ Oliveira et al. recently published a self-paired study in Brazil that showed significant clinical improvement in patients with eosinophilic CRSwNP who received azithromycin, but this group is different than the one that showed better results in the present study and in the literature, with a lower eosinophilic profile.^11^ The future definition of the most prevalent endotype profiles in each geographic region of Brazil will probably help us to better understand this difference in results.

We demonstrated a better outcome for patients with CRSwNP and low IgE, which confirms previously published data. Haruna et al. also showed that there was a worse outcome after macrolide use for patients with extensive nasal polyposis and eosinophilia.^27^ This may be partly explained by the fact that MACs exert an inhibitory effect on neutrophilic inflammation promoters such as Interleukin-8. Suzuki et al., in a study of 16 patients with CRS, also showed symptom improvement associated with low IgE levels and low eosinophil count in peripheral blood, nasal discharge, and nasal mucosa.^27^

In the group that responded to treatment, we did not find a lower number of patients with asthma, rhinitis or AERD. Nevertheless, adults with CRSwNP should be asked about lower airway symptoms and hypersensitivity to acetylsalicylic acid (ASA) and other NSAIDs.

The subgroup with clinical improvement also had elevated IgG and IgM levels in the pre-treatment period, when compared to the other subgroup (both with *p* < 0.01). These data are secondary and further studies may assess the relevance of these data for this group of patients, perhaps related to a possible greater Th1 and lower Th2 population in these patients with normal IgE.

Moriyama et al. showed better subjective and endoscopic results with the addition of long-term low-dose erythromycin in the postoperative period following sinusectomy.^28^ In 2002, Cervin et al. published the first, English-language journal article about the use of a MAC, erythromycin, for CRS. The 500 mg /day dose was administered for 12 months to patients with CRS and the results resembled those published in the Japanese literature.^14^ These studies involved a small number of patients or did not have a control group with placebo.^14^, ^27^, ^28^, ^29^ Ragab et al. published the first prospective, randomized, non-placebo controlled study that compared clinical treatment with erythromycin (1000 mg/day for 15 days, followed by 500 mg/day for 75 days) with surgical treatment for patients with CRS with and without polyposis. Both groups showed improvement in clinical symptoms, nasal nitric oxide measurement, acoustic rhinometry, nasal saccharin clearance time, and nasal endoscopy. All of these were statistically significant and similar, except for the higher total nasal volume observed in patients submitted to surgical treatment.^29^

Wallwork et al. published the first randomized, double-blind, placebo-controlled study of the use of a macrolide, roxithromycin, in patients with CRP without polyposis. A dose of 150 mg/day was administered for 3 months and the patients showed improvement in clinical symptoms, endoscopic evaluation, nasal saccharin clearance time, and nasal IL-8 levels. Similar to previous studies, results were better in patients with low IgE levels.^30^

The key to effectively implementing successful macrolide treatment in CRS is the selection of patients with characteristics known to be associated with a good response to treatment. For this group of patients, CLA treatment could be offered as an alternative to surgery. Another possibility would be postoperative use for patients without preoperative corticosteroid use, in which tissue analysis showed low eosinophilia, as shown by Oakley et al.^31^ However, our study did not show an association between eosinophilia and clinical improvement, differently from that found for low levels of IgE.

This study did not show any adverse reactions or side effects that resulted in medication withdrawal. Side effects associated with the use of erythromycin and roxithromycin, commonly gastrointestinal, are more frequent and there is some risk of hepatotoxicity, due to the higher concentration in that organ. It is the opposite with CLA, that exhibits a higher concentration in the respiratory tract.^32^, ^33^ Thus, CLA would be a more suitable option in the MAC class.

Among the study limitations are the absence of a control group with placebo to allow drawing further conclusions regarding the effects of CLA. Additionally, outcome measures were based on validated subjective questionnaires and scores, depending solely on the patient's assessment. However, as most patients were waiting for a new surgical procedure, if any of them were motivated to answer the quality of life questionnaire incorrectly, their bias would probably be to show less improvement. Another limitation is related to the losses in the group that completed the follow-up within 3 months of treatment completion. We do not know the reason for this; they were simply lost to follow-up. Generally, patients who steadfastly follow-up tend to be those with the best responses to treatment, resulting in a biased sample.

## Conclusion

The use of low-dose clarithromycin for 3 months in patients with recurrent CRSwNP and reduced IgE levels improved quality of life and endoscopic evaluation in this noncontrolled study.

## Conflicts of interest

The authors declare no conflicts of interest.
